# The Sandwich principle: assessing the didactic effect in lectures on “cleft lips and palates”

**DOI:** 10.1186/s12909-020-02209-y

**Published:** 2020-09-15

**Authors:** Anna Bock, Bianca Idzko-Siekermann, Martin Lemos, Kristian Kniha, Stephan Christian Möhlhenrich, Florian Peters, Frank Hölzle, Ali Modabber

**Affiliations:** 1 Department of Oral and Maxillofacial Surgery, University Hospital RWTH Aachen, Pauwelsstrasse 30, D-52074 Aachen, Germany; 2 Audiovisual Media Center, Medical Faculty, RWTH Aachen University, Pauwelsstraße 30, D-52074 Aachen, Germany; 3 Department of Orthodontics, University Witten/Herdecke, Alfred-Herrhausen-Straße 44, D-58448 Witten, Germany

**Keywords:** Sandwich principle, Education, Activating elements, Individual learning

## Abstract

**Background:**

A teaching concept, that takes individual learning and personal belongings into account, is called the “sandwich principle.” This didactic method is an educational concept that alternates consecutively between individual and collective learning phases during a course. This study aimed to prove whether the application of the sandwich principle in lectures increases the learning outcome compared with classical lectures.

**Methods:**

All participants (*n* = 64) were randomly allocated into two groups. One group attended a classical face-to-face lecture and the other attended a lecture that was modified according to the sandwich principle, including activating elements. To compare knowledge gain after the lectures, all the participants had to answer a test comprising40 single-choice questions. In addition, the lectures were evaluated.

**Results:**

Students attending the sandwich lecture had significantly better scores in the test than those who attending the classical lecture (*p* <  0.001). The mean test score of the sandwich group was 63.9% [standard deviation (SD) = 10] points and of the control group 50.2% (SD = 13.7 points). Overall, both the class conditions showed good evaluation results; however, students of the sandwich lecture were more satisfied with the lecture format compared with the other group.

**Conclusion:**

Our study results confirm the thesis that the application of the sandwich principle in lectures increases the learning outcome compared with classical lectures. Even with a big audience, the sandwich design presents a concept that helps maintain high attention levels and addresses individual learning styles.

## Background

Over the last decades, academic teaching has changed in accordance with technological developments and social values [1]. The social values in education demand high topicality, fast information acquisition, integration of learning in daily life, self-organized learning, and independence of time and place [2]. Overall, a student-centered education is required that facilitates the learning process and makes the educational quality more effective.

Learning is a highly individual process that is influenced by multiple factors [3]. Some of these factors are absorption and adaptive capacity, learning speed, and learning style [4–7]. Kolb described learning styles as patterns of behavior based on individuals’ backgrounds and experiences [4, 8]. His learning theory states that the combination of perceiving and processing results in four learning styles: diverging, assimilating, converging, and accommodating. This theory is widely accepted and has been applied in several previous studies [4, 9–12]. Most of these studies underline the diversity of each individual’s learning style [4, 9, 10].

A teaching concept, that takes individual learning and personal belongings into account is called the “sandwich principle.” This didactic method is an educational concept that consecutively alternates between individual and collective learning phases [3, 13]. In general, collective learning phases are passive and most likely similar to classical lectures. The lecturer speaks in front of the class with the students listening passively. In the sandwich principle, the collective learning phase is a compact mediation of knowledge similar to a keynote speech. It supposedly has a maximum duration of 20 to 25 min, which is within the attention span of the students [3, 14].

The individual learning phases are active learning phases. During these phases, students are supposed to repeat, order and apply the previously gained knowledge. This can be achieved via precise work assignments, which activate each student to participate. Partner discussions, partner interviews or small group works are some examples of activating elements. The application of a previously learned lesson helps process the knowledge from short-term to long-term retention. With this alternation of collective and individual learning phases, there is a consecutive switch between passive and active learning.

Another important aspect of the sandwich principle is the entrance and exit of the course. The entrance is supposed to capture the students’ attention and to get them out of the individual phase. Therefore, a meaningful context should be created and learning objectives ought to be pointed out.

The exit of the course should summarize the previously learned lessons. In addition, it serves as a connection between the new knowledgeand its application outside the course.

The sandwich model can be applied to classical lectures, seminars, bedside teaching, or complete courses [3].

The use of the sandwich principle has been promoted in all kinds of teaching to generate high-quality education. However, there has been no study confirming its benefit. Therefore, the presentstudy aimed to prove whether the application of the sandwich principle in lectures increases the learning outcome compared with classical lectures using the example of lectures on cleft lips and palates.

## Methods

### Study design

**Fig. 1:**
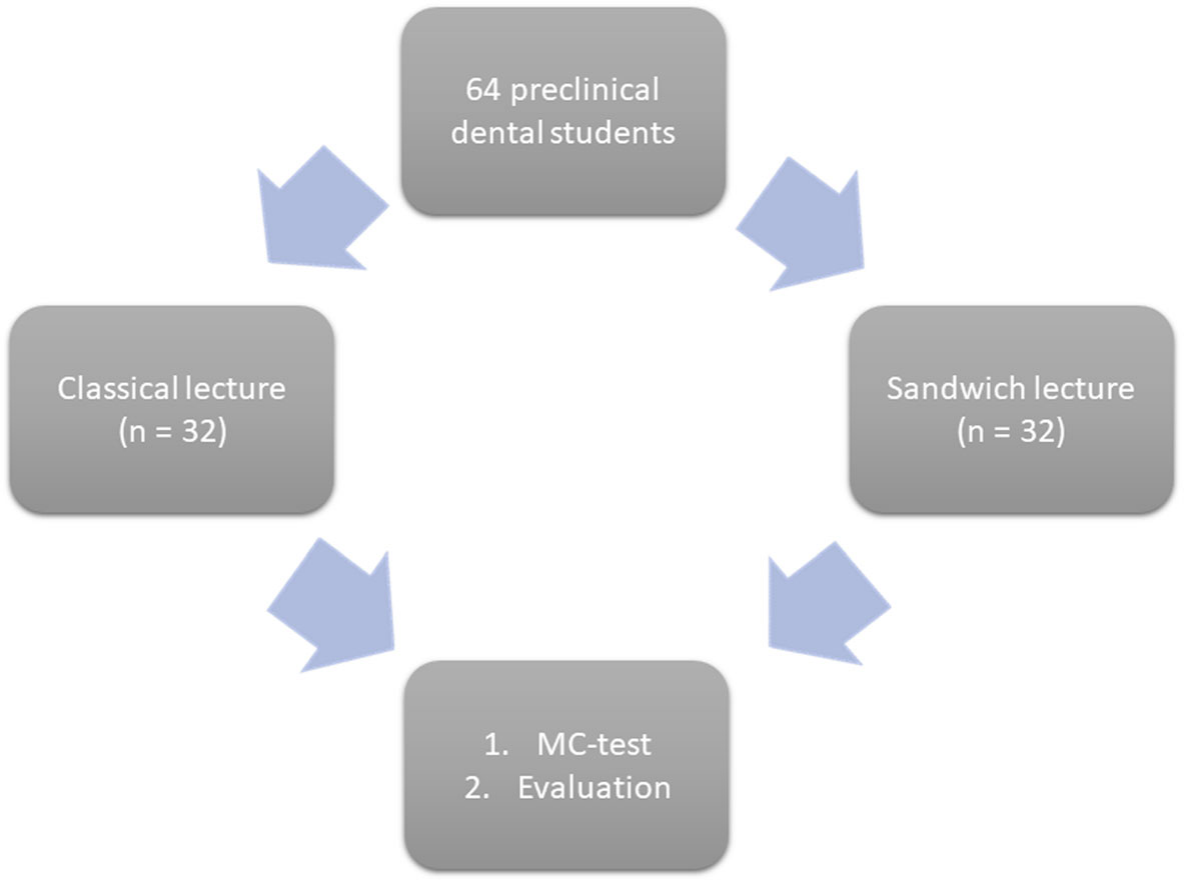
Illustration displaying the study design

Preclinical dental students (*n* = 64), without prior knowledge about cleft lip and palate, were invited to participate in this study. After obtaining informed consent, all the voluntary participants were randomly allocated into two groups. Half of the participants attended a classical face-to-face lecture. The other half attended a lecture modified according to the sandwich principle (Fig. 1). Both the lectures were conducted simultaneously in adjacent lecture halls and lasted 90 min. To compare knowledge gain, all the participants had to answer 40 single-choice questions immediately after the lectures. Additionally, the lectures were evaluated. The need for ethics approval was waived by the institutional review board RWTH Aachen University (EK 137/15).

### Lecture

The topic of the lecture was “etiology and therapy of cleft lips and palates.” The entire content of both the lectures was the same. The classical lecture was in a traditional format where the student is a passive spectator of the lesson. The structure of the sandwich lecture was modified according to the principle. Collective and individual learning phases were integrated alternatively. As an activating element during the individual learning phases, the Tele-Dialog (TED) system was used. The TED system is an electronic real-time voting system for lectures. It is supposed to encourage students to anonymously answer the questions on the lecture and to get immediate personal feedback on their acquired knowledge. Class responses were displayed and discussed in the lecture to enhance the understanding and retention of acquired knowledge. In addition, didactically edited surgery videos were applied as multimedia tools.

To avoid the bias of two different lecturers, the classical and sandwich lectures were delivered by the same professor, filmed ahead of the study, and then projected via beamer onto a projection screen in the lecture halls.

### Test

In the beginning, 50 single-choice questions about cleft lips and palates were created. Only Kprim or type A questions were used. Kprim questions comprise five corresponding statements or options. For each option, students must decide whether it is “true” or “false”. Type A questions comprise five statements as well, but only one option is correct [15]. To validate the test and prove the questions’ discrimination index and difficulty level, 20 dental students (who were volunteers not involved in the main study) were asked to answered the catalog of questions before conducting the main study. Half of these students had prior knowledge about cleft lip and palate, whereas the other half had no prior knowledge.

The discrimination index helps differentiate between a good and bad student. Therefore, the discrimination index value for a question is high if a student answers it correctly and has a high overall score for the test. When the discrimination index value is 0, there is no difference between good and bad students answering the question [16].

The difficulty level always refers to the group being tested. The calculation is based on the reached mean score for a particular question. Easy questions have a low (0.1–0.4) difficulty level and difficult questions a high (0.8–1) difficulty level [16].

With prior validation, too easy and too difficult questions were eliminated so that the final test comprised 40 single-choice questions. Of these questions, 55% were type A and 45% Kprim questions. The distribution of the questions is shown in Table 1. Each correct answer received 1 point. There were no half or minus points. The maximum score of the test was 40 points.

**Table 1:** Distribution of the difficulty level for the final test

	Difficulty level	Number of questions
Easy	> 0.8–1	15
Upper test optimum	0.8–0.6	18
Lower test optimum	0.5–0.4	4
Difficult	< 0.4	3

### Evaluation

Both the lectures were evaluated using a 6-point Likert scale, where 1 denoted “very good” and 6 “unsatisfactory.” All the other aspects were evaluated on a 10-point Likert scale, where 1 indicated “fully agree/very good” and 10 “totally disagree/unsatisfactory.”

### Statistics

The obtained data were arranged using MS Office Excel 2016® (Microsoft Corporation, Redmond, Washington, USA). Statistical analyses were performed using GraphPad Prism 6 Software (GraphPad Software, San Diego, California, USA). An unpaired *t*-test was used for between-group comparison of the test results; the evaluation results of the two groups, after normal distribution, were checked using the D’Agostino-Pearson normality test in omnibus K2 variant. The Pearson correlation coefficient was used to evaluate the results of the TED system with equal questions for both the groups in the final test. Univariate ANOVA was used to proof whether the differences between the groups were related to demographic variables. *P* ≤ 0.05 was considered significant.

## Results

### Participants

All the 64 preclinical dental students were invited to participate in this study voluntarily. On the basis of the academic course of the students, it was assumed that all the participants had no prior knowledge of the topic. Table 2 shows the distribution of the participants.

**Table 2:** Distribution of the participants

	Sandwich lecture (*n* = 32)	Classical lecture (*n* = 32)
Female	78% (25)	81% (26)
Male	22% (7)	19% (6)
19–21 years old	66% (21)	72% (19)
22–24 years old	9% (3)	23% (6)
> 24 years old	25% (8)	5% (7)

### Test

**Fig. 2:**
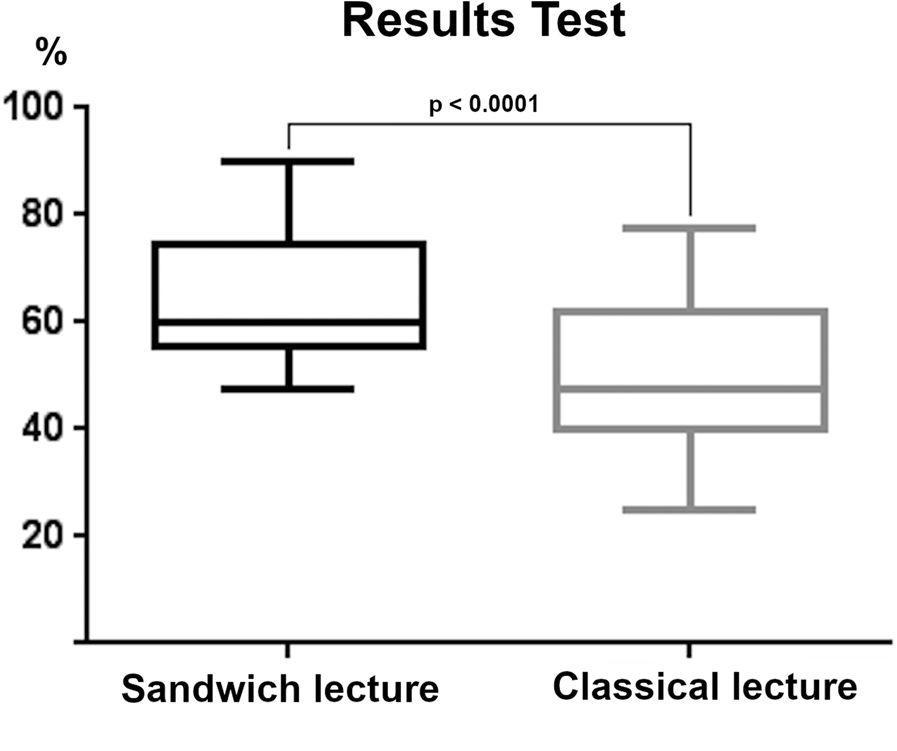
Boxplot of the test results of both the groups in percentage. The group attending the sandwich lecture showed significantly better results than the group attending the classical lecture

The students who attended the sandwich lecture had significantly better scores in the test than those who attended the classical lecture (*p* <  0.0001);Fig. 2). The mean test score of the sandwich group was 25.59 [standard deviation (SD) = 10] points and that of the control group was 16.09 points (SD = 13.7).

The test results are not related to demographic variables (F (2,61) =0.085, *p* >  0.05).

According to the results of the final test, there was a new distribution of the difficulty level for both the groups, as shown in Table 3. For the sandwich lecture group, > 50% of the questions were part of the easy difficulty level, whereasfor the classical lecture group > 50% of the questions were part of thedifficult difficulty level.

**Table 3:** New distribution of the difficulty level of the questions for both groups

	Difficulty level	Sandwich lecture Number of questions	Classical lecture Number of questions
Easy	> 0.8–1	12	7
Upper test optimum	0.8–0.6	10	5
Lower test optimum	0.5–0.4	8	12
Difficult	< 0.4	10	16

### Activating elements

The results of the activating elements, four single choice questions with five answer options each answered using the TED system, are shown in Table 4.

**Table 4:** Results of the TED-questions in the sandwich group

Topic	Correct answer
1. Order of the surgical steps for closure of cleft lips and palates	8.3%
2. Etiology of cleft lips and palates	50%
3. Cleft palates	13.9%
4. Bone grafting in the alveolar cleft	83.9%

No significant correlation was found between the results of the activating elements and the results of the corresponding final test questions with equal learning content (*r =* 0.53; *p* = 0.27).

### Evaluation

The mean scores for the lectures of the sandwich and classical groups were 1.9 (SD = 0.47) and 2.3 (SD = 1.04), respectively (*p* = 0.067). Regarding self-assessment, the students in both the groups showed a significantly better knowledge after the lecture (*p* < 0.0001). The mean score of both the groups was reduced from 7.2 (SD = 2) to 4.0 (SD = 1.56). There was no significant difference in the self-assessment between the two groups.

All the participants in both the groups agreed that they could imagine themselves attending lectures and using videos modified according to the sandwich principle in the future. The mean score for both the groups was 1.76 (SD = 0.88).

The amount of educational content about cleft lips and palates was evaluated as “exactly right” by 82–84% of the participants. Regarding the evaluation of the ability to explain the content of the lecture to another person after attending the lecture, the mean score of the sandwich group was 3.11 (SD = 0.92), whereas that of the classical group was 4.19 (SD = 2.17). There was a significant between-group difference in self-assessment (*p* = 0.0105).

The sandwich group evaluated the activating elements as well. All the results for this part of the evaluation are displayed in Table 5. The majority of the students rated the activating elements as useful. Additionally, the students confirmed that their understanding and attentiveness was raised through the activating elements and that the content of the lecture was better reflected when interacting with the activating elements. They also agreed that the activating elements helped them understand the surgical procedure.

**Table 5:** Evaluation of the activating elements

Aspects	Mean score	Standard deviation
Usefulness of the activating elements	3.12	1.68
Increased of attentiveness and understanding	3.17	1.9
Increased reflection of educational content	2.63	1.53
Understandability of surgical procedure	2.77	1.62

## Discussion

The primary aim of this study was to assess whether the application of the sandwich principle to lectures increases the learning outcome compared with classical lectures. As described by Kadmon et al., the sandwich principle takes students’ requirements into account by considering individual learning styles and attention span. Therefore, it is supposed to increase the efficiency of learning, but so far, there has been no evidence to support this hypothesis [3].

Our study results confirm the assumption that a sandwich-designed lecture is more effective and increases knowledge gain compared with classical lectures. The students attending a sandwich-designed lecture exhibited significantly better scores on the test than those attending a classical lecture. Besides that, the favorable test results demonstrate better attention during the sandwich-designed lecture, confirmingthe thesis that the attention span was considered during the lecture.

In our study, didactically edited surgery videos and TED questions were used to keep the attention level high during the sandwich-designed lecture. A study by Bunce et al. using the TED system confirmed positive effects on attention during the use of activating elements and the part after [14]. Furthermore, Duggan et al. showed high participation during the entire lecture using the TED system; they pointed that the TED system reflects learning difficulties [17]. Regarding our results of the TED questions, only one question was answered correctly by more than 50% participants. Correlating these results with the questions of the final test that had the same content, the number of students answering the questions correctly was higher in the sandwich-designed lecture group than in the control group. This confirms the assumption that the content of the TED questions is reflective and helps to understand the topic better. The results of the TED questions do not help conclude anything about knowledge gain.

Moreover, the activating elements support autonomous learning, thereby promoting a positive learning climate [18, 19]. Our evaluation of the lecture emphasizes the satisfaction of the students with the activating elements. The students pointed that the activating elements helped them reflect the content and improve knowledge comprehension. In addition, the students confirmed that the activating elements increased their attention level and that they would appreciate having activating elements integrated more often. This supports the educational theory that active learning motivates students to learn more [20, 21]. Overall, both lectures showed good evalution results; however the students attending the sandwich lecture were more satisfied with the lecture format compared to those attending the classical lecture.

Compared with other studies, which only captured personal perceptions via evaluation sheets to assess new teaching methods, our study involved objective and subjective evaluations to assess the sandwich principle [3, 22–28]. The objective evaluation was performed using the final test to analyze the knowledge gain. A limitation of our study was that no pretest was conducted to assess baseline knowledge. However, the topic of the lecture was quite complex, and preclinical students have no prior in-depth knowledge about cleft lips and palates in general. This was confirmed by the participants in the self-assessment regarding prior knowledge. Moreover, to overcome the limitation of possibly uneven distribution, the students were equally allocated to both the groups in terms of gender, age, and year of academic course.

In a study by Schönwetter et al., questions of the final test had to be sorted out afterward, because they were answered 100% correctly by both groups that were being compared [29]. To eliminate such limitations in our study, the final test was validated initially by a group of students. Ten students with and 10 students without prior knowledge about cleft lips and palates answered the questionnaire ahead of the main study. The test questions were selected precisely after assessing the discrimination index and difficulty level. Therefore, all questions were informative, and no question had to be eliminated afterward in the final knowledge acquisition test.

According to Kadmon, one disadvantage of the sandwich principle is that less learning material can be presented compared with that in classical lectures [3]. In our attempt to evaluate this new teaching method, completely same content was presented in both the lectures. Therefore, this detriment of less educational content was not approved in our case. Yet, this aspect is comprehensible depending on the complexity and duration of the activating elements used.

Our study could be criticized for the lecture that was filmed ahead and projected on a screen. Therefore, it could be called a “voice-over screen-captured learning” and not as a “face-to-face lecture.” In our opinion, it was more important to overcome the bias of two different lecturers, and there was no other alternative to have the same lecturer simultaneously in two lecture halls. Furthermore, a study by Schönwetter et al. showed that students had comparable learning outcomes irrespective of whether they experienced a face-to-face or an online lecture, i.e.,a “voice-over screen-captured learning.” [29] Other studies have shown similar results [30–32]. However, in our study, long-term retention of knowledge was not measured in our study. This limitation should be addressed in future research.

## Conclusion

Our study confirms the thesis, that the application of the sandwich principle in lectures increases the learning outcome compared with classical lectures. Even with a big audience, the sandwich design presents a concept that helps maintain high attention levels and addresses individual learning styles.

## Supplementary information

**Additional file 1.**

## Data Availability

The datasets used and/or analysed during the current study are available from the corresponding author on reasonable request.
