# Supplementary material for: The Sandwich principle: assessing the didactic effect in lectures on “cleft lips and palates”
Source: BMC Med Educ. 2020 Sep 15;20:310. doi: 10.1186/s12909-020-02209-y (PMC7493973; doi:10.1186/s12909-020-02209-y)
Supplement: Supplementary file 1 — Additional file 1. [file 12909_2020_2209_MOESM1_ESM.pdf]

Evaluation questionnaire

1. In my opinion, the lecture itself was ….

   ☐ ☐ ☐ ☐ ☐ ☐
   1 2 3 4 5 6
   very good unsatisfactory

2. My knowledge on cleft lips and palates **before** attending the lecture was….

   ☐ ☐ ☐ ☐ ☐ ☐ ☐ ☐ ☐ ☐
   1 2 3 4 5 6 7 8 9 10
   very good unsatisfactory

3. My knowledge on cleft lips and palates **after** attending the lecture was….

   ☐ ☐ ☐ ☐ ☐ ☐ ☐ ☐ ☐ ☐
   1 2 3 4 5 6 7 8 9 10
   very good unsatisfactory

4. I could imagine myself participating in lectures and using videos modified according to the sandwich principle in the future.

   ☐ ☐ ☐ ☐ ☐ ☐ ☐ ☐ ☐ ☐
   1 2 3 4 5 6 7 8 9 10
   fully agree totally disagree

5. The amount of educational content about cleft lips and palates was exactly right.

   ☐ ☐ ☐ ☐ ☐ ☐ ☐ ☐ ☐ ☐
   1 2 3 4 5 6 7 8 9 10
   fully agree totally disagree

6. I feel able to explain the content of the lecture to another person after attending the lecture.

   ☐ ☐ ☐ ☐ ☐ ☐ ☐ ☐ ☐ ☐
   1 2 3 4 5 6 7 8 9 10
   fully agree totally disagree

Evaluation of activating elements (only Sandwich-Group)

1. The breaks during the lecture for the activating elements, to reflect the recently learned, are very useful.

   ☐ ☐ ☐ ☐ ☐ ☐ ☐ ☐ ☐ ☐
   1 2 3 4 5 6 7 8 9 10
   fully agree totally disagree

2. My attentiveness and understanding were raised by the activating elements.

   ☐ ☐ ☐ ☐ ☐ ☐ ☐ ☐ ☐ ☐
   1 2 3 4 5 6 7 8 9 10
   fully agree totally disagree

3. The activating elements increased the reflection of the educational content.

   ☐ ☐ ☐ ☐ ☐ ☐ ☐ ☐ ☐ ☐
   1 2 3 4 5 6 7 8 9 10
   fully agree totally disagree

4. The activating elements helped to increase the understandability of the surgical procedures.

   ☐ ☐ ☐ ☐ ☐ ☐ ☐ ☐ ☐ ☐
   1 2 3 4 5 6 7 8 9 10
   fully agree totally disagree
